# The BMP Pathway in Blood Vessel and Lymphatic Vessel Biology

**DOI:** 10.3390/ijms22126364

**Published:** 2021-06-14

**Authors:** Ljuba C. Ponomarev, Jakub Ksiazkiewicz, Michael W. Staring, Aernout Luttun, An Zwijsen

**Affiliations:** Department of Cardiovascular Sciences, Center for Molecular and Vascular Biology (CMVB), KU Leuven, Herestraat 49 Box 911, 3000 Leuven, Belgium; Ljuba.Ponomarev@kuleuven.be (L.C.P.); kubaksiazkiewicz@gmail.com (J.K.); Michael.Staring@gmail.com (M.W.S.); Aernout.Luttun@kuleuven.be (A.L.)

**Keywords:** BMP, BMP pathway fine-tuning, lymphatic vessel biology, mechano-transduction, vascular malformations, signaling cross-talk

## Abstract

Bone morphogenetic proteins (BMPs) were originally identified as the active components in bone extracts that can induce ectopic bone formation. In recent decades, their key role has broadly expanded beyond bone physiology and pathology. Nowadays, the BMP pathway is considered an important player in vascular signaling. Indeed, mutations in genes encoding different components of the BMP pathway cause various severe vascular diseases. Their signaling contributes to the morphological, functional and molecular heterogeneity among endothelial cells in different vessel types such as arteries, veins, lymphatic vessels and capillaries within different organs. The BMP pathway is a remarkably fine-tuned pathway. As a result, its signaling output in the vessel wall critically depends on the cellular context, which includes flow hemodynamics, interplay with other vascular signaling cascades and the interaction of endothelial cells with peri-endothelial cells and the surrounding matrix. In this review, the emerging role of BMP signaling in lymphatic vessel biology will be highlighted within the framework of BMP signaling in the circulatory vasculature.

## 1. Introduction

Dysfunction of endothelial cells lining the inner wall of the circulatory and lymphatic vasculature is a major cause and amplifier of vascular disease. Mutations in genes encoding different components of the bone morphogenetic protein (BMP) pathway cause rare but severe vascular diseases. Most of these diseases are due to loss of function of BMP signaling [1,2,3], but some vascular anomalies also result (indirectly) from a gain of function of BMP signaling [4]. Together, this underscores that the BMP signaling levels need to be well balanced in vascular development and physiology. Nowadays, the BMP pathway is an important therapeutic target for treatment of vascular diseases [3].

In this review, we discuss the important and highly fine-tuned BMP pathway in the context of the endothelium. The major functions of the BMP pathway are first discussed in the high-flow circulatory system, and then emphasis is given to the emerging BMP functions in the low-flow unidirectional lymphatic system. Currently, the role of BMP signaling in the lymphatic system is primarily documented in animal models and human cell cultures. However, it can be expected that, in analogy to the blood vasculature and vascular disease, human lymphatic vascular anomalies resulting from unbalanced BMP signaling are likely to be discovered.

## 2. The Core of the BMP Signaling Pathway

In 1965, the activity of a BMP was first reported by M. Urist, who described that a proteinaceous component in demineralized bone extracts can induce ectopic bone formation when implanted under the skin or into the muscle in different laboratory animals [5]. The protein responsible for this ectopic bone induction was named BMP in 1971 [6]. Since then, BMPs have been extensively studied in bone and cartilage formation, with some BMPs also being used in specific clinical treatments of, e.g., non-union bone fractures [7]. Moreover, germline deletion of several BMP pathway components in animal models results in embryonic lethality [8]. Such studies show that BMP signaling governs very versatile functions during embryonic development and in adult tissue homeostasis, and in nearly every cell type of the body.

We first give an overall view on the core of the BMP pathway (Section 2) and subsequently highlight the key components of the BMP pathway in the vasculature (Section 3). In Section 4, we zoom in on the manifold fine-tuning of this pathway in the vasculature. This review is closed with an overview of vascular diseases due to aberrant BMP signaling.

### 2.1. BMP Ligands

The BMPs belong to the family of Transforming Growth Factor β (TGFβ) secreted growth and differentiation factors [1,9]. As their name indicates, BMPs are morphogens, which means that these ligands can elicit diverse cellular responses in a given cell type depending on the signaling level and duration. Morphogen thresholds contribute to providing positional information to cells within a tissue, i.e., during development or in tissue regeneration [10]. BMPs elicit different cellular responses through their binding to different receptor complexes that activate various intracellular signaling cascades. Based on their sequence similarity and receptor binding affinity, the BMP ligands are subdivided into four subgroups: (i) BMP2 and -4; (ii) BMP5, -6, -7 and -8b; (iii) BMP9 and -10; and (iv) BMP12, -13 and -14 [11].

### 2.2. BMP Receptors

**Figure 1:**
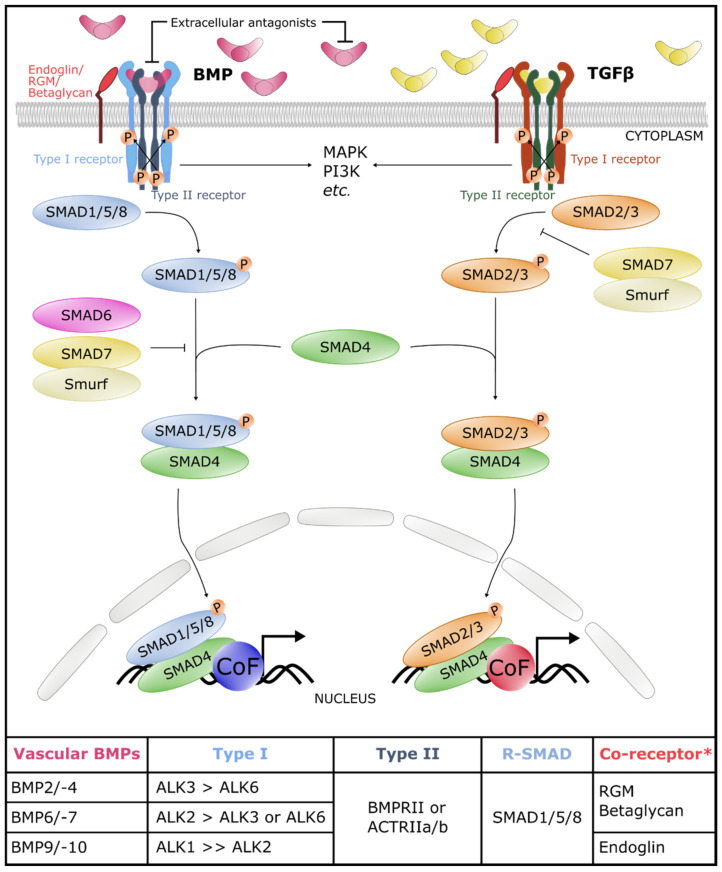
Core of the BMP- and TGFβ-SMAD signaling pathways (details are provided in the text). The lower part of this figure represents the main interaction specificities of the different types of BMP ligands. * Co-receptor binding is not necessary for every ligand–receptor combination. Abbreviations: ACTRIIa/b: activin receptor type-2a/b; ALK: activin receptor-like kinase; BMP: bone morphogenetic protein; BMPRII: BMP Type 2 receptor; CoF: co-factors; MAPK: mitogen-activated protein kinase; P: phosphorylation; PI3K: phosphoinositide 3 kinases; RGM: repulsive guidance molecules; R-SMAD: receptor-regulated SMAD protein; Smurf: SMAD ubiquitin regulatory factors; TGFβ: transforming growth factor β.

BMP ligands bind as a dimer to a hetero-oligomeric receptor complex consisting of two different serine/threonine kinase receptors, the type I and type II receptors [12] (Figure 1). Seven type I receptors have been identified, called the activin receptor-like kinases, ALK1 to -7. The BMP type II receptors include the type 2 BMP receptor (BMPRII), activin receptor type-2a (ACTRIIA) and activin receptor type-2b (ACTRIIB), while the TGFβ type II receptor is TβRII. Different TGFβ family members have different affinities for the type I receptors. More specifically, BMPs activate ALK1/2/3/6, while ALK4/5/7 are predominantly activated by TGFβ, Nodal and activins. However, this is an oversimplification because, for instance, ALK1 is a type I receptor enriched in the endothelium that not only binds BMP9 and -10 but also TGFβ1 [13,14]. The different BMP ligands also show a clear ligand–type I receptor preference: the BMP2/4 subgroup binds preferentially to ALK3 and -6, and the BMP5/6/7/8b subgroup binds to ALK2, -3 and -6, while the BMP9/10 subgroup has the highest affinity for ALK1 [15,16,17,18,19] (Figure 1). Moreover, the presence of a co-receptor such as endoglin or repulsive guidance molecules (RGM) can enhance the affinity of a BMP ligand for its receptor complex [20,21].

Upon BMP–receptor binding, the constitutively active type II receptor phosphorylates the serine and threonine residues in the glycine–serine-rich (GS) domain of the type I receptor [22]. This triggers downstream canonical or non-canonical signaling, depending on the lateral mobility of the BMP receptors [23].

### 2.3. Canonical and Non-Canonical Signaling Cascades

The canonical or SMAD signaling pathways are the most extensively documented cascades (Figure 1). The name SMAD unites the names of the first proteins of the SMAD family that were discovered in *C. elegans* and *D. melanogaster*, respectively, named “Sma” and “Mother Against Decapentaplegic”. Phosphorylation of the type I receptor results in recruitment of the receptor-regulated SMAD effector proteins or R-SMADs. Depending on the type I receptor that is activated, a different subset of R-SMADs is recruited. Activation of the BMP-specific type I receptors ALK1/2/3/6 phosphorylates the so-called BMP-SMADs SMAD1/5/8. Activation of the TGFβ/Nodal/Activins-SMADs SMAD2/3 results from phosphorylation by the type I receptors ALK4/5/7. However, on some occasions, TGFβ can also activate SMAD1/5/8 complexes [16].

Receptor-mediated phosphorylation of the C-terminal Ser-X-Ser motif of R-SMAD monomers (pSMAD) triggers activation and complex formation between pSMADs and the common SMAD4. This complex is then translocated into the nucleus where it regulates in association with various co-factors (CoF) context-specific pathway-dependent gene expression [24] (Figure 1).

SMADs are subject to numerous post-translational modifications that regulate their activity and allow other signaling pathways to influence the pathway [25]. An example is the phosphorylation of activated R-SMADs by glycogen synthase kinase 3β (GSK3β) and mitogen-activated protein kinase (MAPK), and proteasomal degradation through SMAD ubiquitin regulatory factors-1 and -2 (Smurf-1,-2), which contribute to determining the stability of (activated) SMAD proteins [26,27] (Figure 1). As SMADs show a low affinity for DNA binding, the aforementioned CoFs contribute to regulating transcription by strengthening the interaction between SMADs and DNA and/or changing their activation into repression. In addition to regulating gene expression, SMADs also function in the direct regulation of microRNA (miRNA) maturation and chromatin remodeling [28,29].

In addition to the canonical SMAD-dependent pathways, ligand–receptor activation can also trigger non-canonical intracellular effector phosphorylation. Examples of such non-SMAD pathways include MAPK and Phosphoinositide 3 (PI3) kinases/protein kinase B (AKT) [15,20,30] (Figure 1). These non-canonical pathways have been less well explored in the context of BMP signaling, mainly because these kinases are commonly activated by other signaling pathways, which may confound interpretation of results.

## 3. The Core BMP Signaling Pathway in Blood and Lymphatic Vasculature

In this section, the function of BMP signaling components in the blood vasculature is summarized briefly, owing to excellent recent reviews [1,2,3,4,31]. Conversely, there is growing evidence from studies in mice and zebrafish that the BMP pathway is also critical in lymphatic vessel development and function; however, these studies have not been extensively reviewed. Therefore, we discuss these preclinical animal models and in vitro models in more detail (summarized in Table 1).

### 3.1. BMP Ligands in Blood and Lymphatic Vasculature

**Blood vasculature**—BMP2, -4, -6, -9 and -10 are the BMPs with vascular functions (Figure 1). The functions of these ligands in the endothelium have often been reported first in a tumor setting [14,39,40,41,41,42,43], but later studies also showed their role in vessel development and homeostasis, extensively reviewed in [1,2,3,31]. Overall, BMP2 and -4 are considered locally produced paracrine ligands, whereas BMP6/9/10 are present in the systemic circulation. In general, BMP2/4/6 signaling elicits a pro-angiogenic response, while BMP9/10 signaling inhibits sprouting and contributes to vessel stabilization and quiescence in vascular endothelial cells [1,2,3,31].

**Lymphatic vasculature**—BMP6 and BMP9 are present in the systemic circulation and may thus elicit luminal signaling in lymphatic endothelial cells when taken up as lymph from extravasated fluid. Other BMP ligands, such as BMP2 and BMP4, are more likely to be produced in the vicinity of lymphatic endothelial cells and signal in a paracrine fashion.

BMP9 is essential for lymphatic vessel maturation and especially lymphatic valve formation [33,34]. *Bmp9* knockout (KO) neonates have dilated collecting and capillary lymphatic vessels, and a reduced number of intraluminal lymphatic valves in collecting vessels. BMP9 inhibits *Lymphatic Vessel Endothelial Hyaluronan Receptor 1 (Lyve1)* expression through ALK1 in mesenteric lymphatic vessels which promotes the maturation of the mesenteric lymphatic vessels. Moreover, BMP9 induces the expression of *Forkhead box protein C2 (Foxc2)*, *Connexin37 (Gja4)*, *Ephrin-b2* (*Efnb2*) and *Neuropilin1 (Nrp1)* in an ALK1-dependent manner. These genes are all master genes involved in lymphatic valve formation in mice [33], and with mutations in *FOXC2* causing human lymphatic vascular anomalies [44]. Confirming the results in *Bmp9* KO neonates, adult *Bmp9* KO mice show similar lymphatic capillary and collecting vessel maturation deficits and have inefficient drainage of interstitial fluid [34]. Yoshimatsu et al. also investigated BMP9/ALK1 signaling in BMP9-KO embryos and ALK1-depleted neonates [34]. The ALK1-depleted neonates and embryos at embryonic day (E)15.5 show a dilated lymphatic vasculature, comparable to the BMP9 KO neonates reported by Levet et al. [33]. Interestingly, adenoviral BMP9 administration in a mouse model of chronic aseptic peritonitis and BMP9-expressing breast carcinoma cells inoculated into immunocompromised mice show that BMP9 inhibits lymphangiogenesis, providing an interesting therapeutic option. Moreover, BMP9 downregulates *Prospero Homeobox 1 (PROX1)* expression through ALK1 in human dermal lymphatic endothelial cells (HDLECs), thereby altering cell cycle-related genes, which leads to a restricted HDLEC cell proliferation. Interestingly, the BMP9-mediated downregulation of PROX1 also results in a trans-differentiation of lymphatic endothelial cells to blood endothelial cells [34].

Subileau et al. showed the morphogen properties of BMP9 in lymphangiogenesis in mouse embryonic stem cell differentiation experiments [35]. A low dose of BMP9 causes an expansion of the LYVE1-positive early lymphatic-specified endothelium, while a high dosage of BMP9 expands the LYVE1-negative early lymphatic-specified endothelium. Given that BMP9 is expressed from E10 onwards in the mouse embryo, the authors suggested that it may act recurrently in mouse lymphatic endothelial cell development [45]. First, BMP9 would act as a more pro-lymphatic-vasculogenic factor during the initiation of lymphatic development, while it promotes lymphatic vessel maturation and valve formation later [35].

In addition to BMP9, BMP2 signaling was shown to negatively regulate lymphatic vessel development in zebrafish and mice in a SMAD- and miRNA-dependent manner [32]. BMP2 gain of function inhibits *Prox1* expression in zebrafish, which impedes zebrafish lymphatic vessel development. The BMP2-mediated repression of *PROX1* was confirmed in HDLEC cultures, similar to the BMP9-mediated downregulation of *PROX1* reported by Yoshimatsu et al. [34]. Moreover, BMP2 induces expression of *miR-31* and *miR-181a* in a SMAD-dependent manner. Expression of *miR-31* and *miR-181a* is present in vascular endothelial cells in the cardinal vein, but not in lymphatic endothelial cells that are budding from the cardinal vein. The authors postulated that these miRNAs target *Prox1* and thus restrict lymphatic endothelium specification in the cardinal vein. Additionally, co-administration of BMP2 and VEGF-C in a lymphatic differentiation model using mouse embryoid bodies showed that BMP2-SMAD1/5/8 signaling inhibits the normally VEGF-C-mediated lymphatic endothelial cell induction in the periphery of the embryoid body. Interestingly, in the developing embryo, pSMAD1/5/8 activity was detected in vascular endothelial cells in the cardinal vein, although not in the budding lymphatic endothelial cells, similar to the expression of *miR-31* and *miR-181a*. This study shows that the BMP2 function in the lymphatic vasculature is conserved over different vertebrates [32].

A recent single-cell (sc)RNAseq study to investigate lymphatic endothelium specialization in mouse lymph nodes revealed that several BMP ligands and target genes of BMP signaling are differentially expressed within the lymphatic endothelium of lymph nodes. For instance, the lymphatic endothelium that lines the floor of the subcapsular sinus expresses *Bmp2*, *Bmp6* and *Smad6/7*, while cells lining the ceiling express *Bmp4* [46]. This fuels the hypothesis that BMP signaling fulfills distinct functions in niche-specific specialization of the lymphatic endothelium in lymph nodes.

### 3.2. BMP Receptors in Blood and Lymphatic Vasculature

**Blood vasculature**—The pro-angiogenic BMPs BMP2/4/6/7 signal mainly via the ALK2, ALK3 and ALK6 BMP type I receptors, in conjunction with either BMPR2 or ACVRIIs type II receptors in the endothelium. The anti-angiogenic BMPs BMP9/10 induce signaling via ALK1, the most abundant type I receptor in endothelial cells. The ligand–receptor complexes in the blood vasculature are reviewed in [1,2,3,31] (Figure 1).

**Lymphatic vasculature**—Niessen et al. showed that not only human umbilical vein endothelial cells (HUVECs) but also HDLECs express the BMP type I receptor *ACVRL1* encoding ALK1, *ACVR2B* (ACTRIIB), *BMPRII* and *ENG*, and concomitantly that HDLECs respond to BMP9/10 in an ALK1-dependent manner [36]. The impact of ALK1 in lymphatic development and remodeling was further established by in vivo blockage of ALK1 in neonatal mice, using decoy soluble receptors or ALK1-neutralizing antibodies. This revealed that ALK1 is necessary for the formation and further remodeling of the initial lymphatic plexus in different organs in neonatal mice. Interestingly, using decoy receptors of ACTRIIB or BMPRII resulted in a comparable but less severe phenotype than the ALK1-decoyed or -neutralized neonates. Moreover, chyle accumulation was observed in the ALK1-decoyed neonates, suggesting a defective intestinal lymphatic vasculature in lacteals. However, the authors concluded that this likely results from stalled lymphangiogenesis rather than regression of lymphatic capillaries. Indeed, no regression but a failed remodeling in the honeycomb lymphatic structure of the tail was observed when an ALK1 decoy receptor was administered at later neonatal stages. Moreover, also genetically modified mice with a deficiency in ALK1 show increased lymphatic endothelial cell proliferation together with lymphatic vessel enlargement, similar to *Bmp9* KO embryos and neonates compatible with the scenario that BMP9 predominantly acts through ALK1 to restrict lymphangiogenesis [34].

Zebrafish studies have shown the involvement of the type I receptors Alk3/Alk3b and the type II receptors Bmpr2a/Bmpr2b in lymphatic development. Morpholino anti-sense oligonucleotides were used to examine the effects of silencing of these BMP signaling components on lymphatic development. In contrast to the anti-lymphangiogenic effect of Bmp2 in zebrafish [32], reduction in Bmpr2a/b or Alk3/Alk3b causes loss of lymphatic endothelial cells in the thoracic duct. This discrepancy can be explained by either a pro-lymphangiogenic role of other BMP ligands than Bmp2 using these same receptors, by other signaling pathways or by an indirect effect on lymphatic endothelial cells by morpholino-mediated gene silencing in the venous endothelial cells. Interestingly, only silencing of *Smad5*, but not of *Smad1* or *Smad9*, resulted in reduced numbers of lymphatic endothelial cells, suggesting that only Smad5 is indispensable in zebrafish lymphangiogenesis [37].

### 3.3. Canonical and Non-Canonical Signaling Cascades

**Blood vasculature**—SMAD1/5/8 activity, monitored by the presence of nuclear pSMAD1/5/8 proteins, has been described in numerous vascular beds in several studies. Moreover, BMP signaling via SMAD1/5 has been shown to be essential for stalk cell identity during sprouting angiogenesis in mouse embryos [47]. Stalk cells trail the tip cell in sprouting angiogenesis and elongate the angiogenic sprout. Most SMAD1/5 functions seem to be critical during blood vessel development; conversely, the highly similar BMP effector SMAD8 (gene *SMAD9*) seems to function especially during pulmonary vascular homeostasis in adult mice [48] and humans [29,49], illustrating non-redundant roles between SMAD1/5 and SMAD8 in the endothelium. Target genes of SMAD1/5 in endothelial cells are, amongst others, *Smad6/7*, *Atoh8* encoding atonal basic helix-loop-helix transcription factor 8, *Tmem100* encoding transmembrane protein 100, *EGFL7* encoding epidermal growth factor-like domain 7 and *Id*-genes encoding inhibitors of differentiation proteins [9,24,50,51,52,53,54,55], whereas SMAD8 induces *miR-21* and *miR-27a* and suppresses vascular endothelial growth [29]. The non-SMAD BMP pathway is less well understood in the blood vasculature, but BMP2-induced p38-heat shock protein 27 (HSP27)-dependent cell migration promotes tip cell competence and migration [15].

**Lymphatic vasculature—**Many BMP reporter mice have been generated to investigate the transcriptional activity of BMP-SMAD signaling [56,57,58,59,60,61]. One of these BMP reporter mice is the BRE:gfp reporter mouse. Here, the BMP response element (BRE) derived from the ID1 promotor drives the expression of enhanced green fluorescent proteins (eGFP) to track SMAD1/5/8 activity [56]. Beets et al. described the dynamic and spatiotemporal BMP-SMAD activity in the blood and lymphatic vasculature in mouse embryos and in neonatal tissues [62]. Mosaic GFP localization patterns are present in different regions of the developing vascular tree and heart. At E12.5, BMP-SMAD signaling is present in endothelial cells of the cardinal vein and lymphatic endothelial cells budding thereof. This agrees with the low reporter activity that Dunworth et al. reported in budding lymphatic endothelial cells but contrasts with the absence of nuclear pSMAD1/5/8 in these cells [32]. It is most likely that this discrepancy is due to the longer stability of the GFP reporter protein compared to pSMAD1/5/8.

Moreover, dermal embryonic lymphatic vessels at E14.5 and E16.5 displayed a prominent and widespread GFP pattern. In postnatal tissues, GFP-positive lymphatic endothelial cells were mainly confined to the valve-forming regions in collecting vessels [62]. This observation agrees with the previously described role of BMP9 in lymphatic valve formation [33].

Interestingly, increased numbers of lymphatic progenitors and enlarged and blood-filled lymphatics are present in an endothelial-specific KO of *Tmem100*—a target gene of the BMP9/ALK1 axis in vascular endothelial cells. Proliferation and apoptosis are unaffected in the lymphatic endothelium. Conversely, endothelial-specific overexpression of TMEM100 results in reduced lymphatic endothelial progenitors, and a small size and number of disorganized lymphatics. This is compatible with a role of TMEM100 in restricting—just like the Notch pathway and putatively (partially) through Notch signaling—the specification of the lymphatic endothelial cell lineage [38]. This study suggests that TMEM100 is an important downstream effector of BMP9 and ALK1 in the early stages of lymphatic vessel development.

## 4. Fine-Tuning Mechanisms of BMP Signaling in the Vasculature

**Figure 2:**
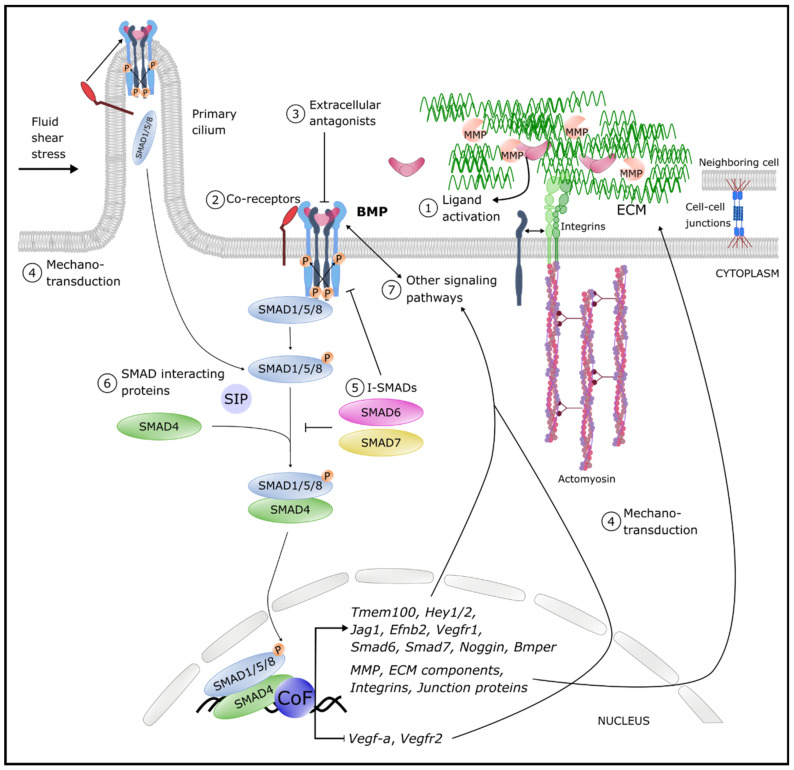
Overview of the different levels of BMP pathway fine-tuning. Circled numbers denote examples of levels of regulation of the signaling output. Cell–cell junctions are tight, adherence and gap junctions (details are provided in the text). Abbreviations: BMP: bone morphogenetic protein BMPER: BMP endothelial cell precursor-derived regulator; CoF: co-factors; P: Phosphorylation; ECM: extracellular matrix; Ephb2: Ephrin B2; Hey: hairy/enhancer-of-split related with YRPW motif protein; Jag: Jagged; MMP: Matrix metalloproteinases; SIP: SMAD interacting proteins; Tmem100: transmembrane protein 100; Vegf: vascular endothelial growth factor; Vegfr: VEGF receptor.

The shaping of BMP morphogen gradients or responses depends on the bioavailability of (tissue-specific) BMP ligand–receptor complexes, and intracellular effectors (Figure 1). In addition, the BMP signaling pathway is further fine-tuned by different extracellular and intracellular agonists and antagonists that bind and sequester BMPs or signaling components. In this respect, it is striking how target genes of BMP signaling often function as negative feedback regulators of BMP signaling themselves. Moreover, the BMP pathway cross-talks with mechanical cues in bone, a feature that is increasingly being recognized in the vessel wall as well [31]. However, also its interactions and cross-talk with other pathways contribute to the contextual status that regulates and fine-tunes the BMP pathway (Figure 2). Here, we provide the most relevant pathway tuning and interplay between BMP signaling and other vascular pathways. These have especially been documented in the blood vasculature and may inspire lymphatic studies of the future.

### 4.1. Ligand Activation

**Blood vasculature**—BMPs are just like other TGFβ members synthesized as large pre-proproteins comprising a signal peptide, a large pro-domain and a mature growth factor domain and then processed as a mature disulfide-linked dimer. For a BMP to become active, dimerization is a prerequisite. Thereto, its pre-proprotein is cleaved by subtilisin-like proprotein convertases, such as Furin; thereafter, the two pro-domains come together to fold and form a covalently bound dimer [63,64,65]. Dimers can bind to the receptor complex initiating downstream signaling.

The pro-domain is an important regulator of latency of TGFβ family members, since the pro-domain can block binding to the receptor complex or affect interaction with the extracellular matrix (ECM) through fibronectin or fibrillin assemblies [64,66]. The TGFβ ligand is ultimately activated when the pro-domain is “stripped off” by tensile forces generated by integrins such as αvβ1, αvβ6 and αvβ8 present on endothelial cells [66,67]. Interestingly, most secreted BMPs are still covalently bound to their pro-domain, therefore they do not exhibit a latency effect and are compatible with receptor binding [68,69]. This was further confirmed by the crystal structure of the non-latent BMP9 and its pro-domain [70].

Mature BMP7 interacts in mice with the fibrillin2 component of the fibrillin assemblies that are present in the ECM of endothelial cells [71]. Moreover, in vitro studies have shown that also different BMP pro-domains can bind to these fibrillin assemblies [65,72]. Furthermore, many BMP ligands can interact with the ECM, heparins, tenascin-c and laminin [71,73,74,75] (Figure 2; number 1). The release of bioactive BMPs to endothelial cells is most likely not integrin-dependent but achieved through a cyclic stretch and strain-dependent secretion of matrix metalloproteinases (MMP) that leads to ECM disruption and BMP ligand release [76,77].

“Mixed” BMPs, achieved by pro-domain heteromerization, are another important diversification of BMP signaling, potentially also in the context of vessels. Recombinant BMP2/5, BMP2/6, BMP2/7 and BMP2b/7 heteromers have been shown to more potently activate the signaling pathway that induces dorsoventral patterning in zebrafish embryos than the respective homodimers [78,79]. Additionally, in mice, endogenous BMP7 functions predominantly as a heterodimer with BMP2 or BMP4 in embryogenesis [80]. These studies reveal that different type I receptors are then recruited into BMP signaling complexes.

**Lymphatic vasculature**—BMP9 and BMP10 are synthesized as large pro-domain-associated precursors that freely circulate in the blood stream while still being associated with their pro-domains. This association does not hamper receptor binding [42], and hence activation of these ligands seems not necessary in the lymphatic vasculature. Conversely, BMP2 and BMP4 are likely to be produced and processed locally in the vicinity of lymphatic endothelial cells and may be trapped in fibrillin assemblies that are present in the anchoring filaments of capillary lymphatic endothelial cells [81,82]. It is tempting to speculate that changes in interstitial pressure may affect their release and confer signaling to the lymphatic endothelial cells; however, this remains to be investigated.

### 4.2. Co-Receptors

**Blood vasculature**—Binding of BMPs with low type I and/or type II receptor binding affinities can be enhanced by the engagement of a co-receptor in the complex, leading to an increased diversity in ligand–receptor assembly [83]. Here, we describe a few examples of different BMP co-receptors with a function in the cardiovascular system (Figure 2; number 2).

Endoglin (CD105) is a type III accessory receptor abundantly, but not exclusively, expressed by activated vascular endothelial cells [84,85], with high affinity for BMP9/ALK1 and lower affinity for TGFβ1/ALK1, and TGFβ1 and -3 in association with ALK5/TβRII [86]. Moreover, endoglin can also bind BMP2 and BMP7 with low affinity [84]. Interestingly, endoglin also mediates a cooperation between fibronectin/α5β1 integrin and TGFβ/BMP signaling in endothelial cells. This cooperation leads to an enhancement of TGFβ1 and BMP9 ALK1/SMAD1/5/8-dependent signaling, ultimately regulating angiogenesis [87].

Betaglycan is another glycoprotein type III accessory receptor which interacts with TGFβ2 but also with BMP2, -4 and 7, enhancing the further downstream pathway. Moreover, it has been shown that betaglycan is essential in endothelial-to-mesenchymal transition (EndMT) in the developing mouse heart through BMP2 signaling enhancement [88,89].

Another type of co-receptor are the glycosylphosphatidylinositol (GPI)-anchored receptors, called the RGMs. These co-receptors sensitize cells for BMPs present at low concentrations, but such activities have not yet been reported in the endothelium [90,91,92].

Interestingly, NRPs and vascular endothelial (VE)-cadherin are important co-receptors for vascular endothelial growth factor (VEGF) signaling, which also interact with BMP/TGFβ signaling receptors. NRP1 and NRP2 bind TGFβ, ALK1/5, the type II receptors and betaglycan, affecting TGFβ ligand sensitivity and sprouting angiogenesis [93,94,95]. VE-cadherin—a key adherence junctional protein in endothelial cells that regulates, amongst others, vascular permeability—retains VEGFR2 at the plasma membrane and enforces VEGF signaling [96]. A similar co-receptor role was established for VE-cadherin in ALK1- and ALK5-mediated TGFβ signaling, potentiating its antiproliferative and antimigratory responses [97]. Moreover, VE-cadherin can also associate physically—in a BMP6-dependent manner—with ALK2 and BMPRII, which stabilizes the BMP receptor complex for further downstream signaling in endothelial cells [98].

**Lymphatic vasculature**—It is likely that the BMP pathway is tuned in the lymphatic endothelium by similar NRP1-, NRP2- or VE-cadherin-mediated interactions with the BMP receptor complex to those in the blood vasculature (previous paragraph), but hard evidence is still lacking.

### 4.3. Antagonists

**Blood vasculature**—The BMP antagonists (Figure 2; number 3) are classified into three classes based on their structure: the Chordin/Noggin family, Twisted/Gastrulation (TWSG1) and the DAN/Cerberus family (which includes Gremlin1 (GREM1)) [99].

Noggin antagonizes BMP signaling by binding to BMP2/4 with high affinity and BMP7 with low affinity, while BMP9/10 are not bound by Noggin [100]. Gain-of-function studies with Noggin have demonstrated the role of certain BMP ligands in, e.g., the development of the cushion endocardium [101] or in embryonic blood vessels in the quail, where Noggin inhibits BMP4 activity induced by VEGFR2 [102]. Noggin is endogenously induced in the bone endothelium by Notch-mediated signaling during endothelial cell proliferation and vessel growth in the skeletal system [103]. Noggin functions in the bone primarily as an angiocrine factor that promotes chondrocyte maturation and hypertrophy, which in turn affects angiogenesis through *Vegf-a* expression. The signaling interactions between Notch, BMP and VEGF signaling pathways couple angiogenesis and osteogenesis in the bone [103].

GREM1 binds to BMP2/4/7 with high affinity, blocking ligand–receptor interaction [104]. In mice, *Grem1* haplodeficiency results in a pulmonary hypertension phenotype [105]. Additionally, increased expression of GREM1 was observed within the endothelial cell layer of lung tissue obtained from patients with idiopathic and hereditary pulmonary hypertension. GREM1 also mediates EndMT in pulmonary arterial endothelial cells, and this EndMT can be reverted by BMP7 [106]. Interestingly, GREM1 also functions in a BMP ligand-independent manner [104,107,108]. For instance, GREM1 can bind to endothelial cells, activating multiple pathways that affect extracellular signal-regulated kinase (ERK), paxillin and focal adhesion kinase (FAK). As such, GREM1 co-regulates migration and matrix remodeling by endothelial cells in a BMP-independent manner [109].

Some BMP antagonists can also bind with each other, thereby potentiating the inhibition of BMP signaling. For example, Noggin and GREM1 interact and cooperate in clathrin-dependent endocytosis of BMP in endothelial cells. This reduces the duration and magnitude of BMP4-dependent SMAD signaling [110]. Likewise, the BMP modulators BMP endothelial cell precursor-derived regulator or BMPER (also called crossveinless-2 or CV2) and TWSG1 cooperate in HUVEC sprouting both in vitro and in vivo [111,112,113]. Such a cooperation between Bmper and Twsg1 has been shown in zebrafish embryos to be crucial for the preservation of the arterio-venous specification through the specific regulation of the Notch signaling pathway [114]. Moreover, BMPER exerts endothelium protective functions and antagonizes tumor necrosis factor α-induced vascular inflammation [115].

BAMBI (BMP and Activin membrane bound inhibitor) is expressed on endothelial cells and acts as a non-signaling, competitive antagonist of type I receptors such as ALK 1 and -5. Deficiency of BAMBI in mice results in an unusual arterial wall neovascularization that surprisingly mimics features of intra-plaque hemorrhage of advanced atheroma in a mechanical injury model. This is compatible with a role of BAMBI in arterial endothelial cell homeostasis [116].

R-spondins are secreted co-activators of WNT signaling with functions in the vascular [117] and the lymphatic endothelium [118]. Interestingly, R-spondins have recently been shown to inhibit BMP signaling in a WNT-independent fashion in early *Xenopus* embryos [119]. Confirmation of a similar function in the vessel wall remains to be demonstrated.

**Lymphatic vasculature**—The above-described BMP antagonists have thus far not been described to modulate BMP signaling in the lymphatic vessel wall.

### 4.4. Mechano-Transduction

**Blood vasculature**—The importance of mechano-transduction in the (dynamic) regulation of endothelial phenotypes and features is well appreciated in vessel biology [120,121]. Mechanobiological cues involve wall shear stress, elicited by flow or strain, and tension forces from the ECM and the surrounding cells. These forces are integrated by the cells via the dynamic interactions between, amongst others, integrins, junctional proteins, cytoskeletal rearrangements and growth factor receptors [120]. Interestingly, mechanical cues and BMP signaling cooperate in vascular endothelial cell biology and function in several ways, as earlier established in bone biology [31,121] (Figure 2; number 4).

Responses of vascular endothelial cells to fluid hemodynamic forces from the blood flow govern the development, physiology and diseases of vessels. These responses already occur during the initial development of the vascular plexus, when hemodynamic mechanical stimuli and growth factors contribute to the patterning and remodeling of the plexus [122]. Indeed, multiple pathways including the Notch, WNT, VEGF and BMP/SMAD pathways contribute to integrating fluid shear stress (FSS) that controls arteriovenous differentiation, cell rearrangements and vascular remodeling. Mature vessels are also subject to hemodynamic forces that affect endothelial cell turnover and homeostasis. High laminar flow in straight vessel segments provides resistance to atherosclerosis, while lower flow and disturbed flow sensitize the vessel to inflammatory pathways and atherosclerosis. Typically, values for FSS in healthy vessels range from highly pulsatile 1–4 pascals (Pa; 10–40 dynes/cm^2^) in arteries to 0.1–0.6 Pa with low pulsatility in veins [120]. Interestingly, the expression of some BMP signaling components, such as *BMP4*, *Noggin* and *Grem1*, is regulated by flow. For instance, *BMP4* is strongly downregulated in laminar shear stress conditions in vascular endothelial cells, whereas it is induced under disturbed flow conditions [123]. Moreover, recently, it has been shown that Notch1-mediated upregulation of SMAD6 is necessary for flow-mediated alignment, homeostatic quiescence and the barrier function of blood endothelial cells [124].

An intriguing mechanism by which mechanical forces can affect the BMP pathway is how such ligands are made bioavailable. As described in Section 4.1, several TGFβ/BMP ligands, but also antagonists such as Noggin, bind to different components of the ECM and the glycocalyx, a thick coat of glycoproteins and proteoglycans. Tensile forces generated by specific integrins or expression of MMPs can release and activate the trapped ligands from this reservoir [66,67,71,73,74,75,76,77,125,126,127]. In addition, the stiffness of the ECM also affects vessel development and health. For instance, ECM stiffness is important for blood capillary maintenance [128]. In arteries, BMP2 expression positively correlates with the stiffness of the ECM [129]. Moreover, an ECM with high stiffness is observed in the TGFβ-mediated EndMT in aortic valve endothelial cells [130].

Another mechanism of how mechanical cues tune BMP signaling is through colocalization of integrins and focal adhesion components with BMP receptors and the internalization of the BMP receptors [131]. As mentioned in Section 4.1, ALK1 and its co-receptor endoglin colocalize with α5β1 integrin to induce angiogenesis [87]. Interestingly, α5β1 integrin acts as a switch in cancer cell lines between the cytoskeleton and ECM mechanics to influence adhesion-dependent motility and different signaling pathways [132]. Moreover, disturbed shear stress induces an interaction between BMPRII and α5β3 integrin to activate SMAD1/5/8 through an Shc/FAK/ERK pathway, resulting in vascular endothelial cell cycle progression [133,134].

Junctions in vascular endothelial cells are another site in the cell membrane where mechano-transduction and BMP signaling interact. For instance, the gap junction protein connexin37 has recently been described to be differentially regulated upon mechanical stimulation of SMAD1/5/8 signaling in endothelial cells [135]. Additionally, loss of connexin40 is shown to increase the formation of arteriovenous malformation in an ALK1-dependent manner [136]. The adherence junction molecule VE-cadherin and its cooperation with the BMP signaling pathway have already been described in Section 4.2.

The luminal primary cilium is a signaling hub that integrates, amongst others, flow-derived signals. The BMP/ALK1 axis is specifically enriched at the primary cilium, and prominent phosphorylation of SMAD1/5/8 is observed at the basal body and along the cilium. Furthermore, FSS increases the physical interaction between ALK1 and endoglin and thereby lowers the effective concentration of BMP9 required for ALK1 activation. The cilium thus sensitizes blood endothelial cells to BMP9 ligands and prevents excessive vascular regression [137].

Interestingly, the intracellular actin cytoskeleton and mechanosensory components of the nucleus also affect BMP signaling output and vice versa. For example, actin-driven filopodia and cytoskeletal rearrangements are vital for the leading tip cell in angiogenic sprouts [138]. BMP6 receptor activation of non-SMAD pathways, such as MAPK-p38-HSP27 signaling, stimulates endothelial cell migration by rearranging the cytoskeleton [15]. Another study showed that Myosin-X (Myo10) is a BMP6 target gene in vascular endothelial cells implicated in cellular alignment and directional migration. Strikingly, Myo10 and the BMP6 receptor ALK6 colocalize and translocate into filopodia after BMP6 stimulation [131].

**Lymphatic vasculature**—The lymphatic vasculature is a low-flow system (up to 0.6 Pa) with a very high pulsatility [120]. The lymphatic endothelium is also very sensitive to changes in mechano-transduction. In general, it has been shown that lymph flow and different flow patterns contribute to the maturation, patterning and stabilization of the lymphatic vessels into capillaries and collectors [139,140]. Collecting vessels become patterned, amongst others, by flow into lymphatic valve regions and lymphangions, the contractile vessel segments between two valve regions. In the development of the lymphatic system, ECM stiffness has a strong impact; a soft ECM is essential for lymphatic vessel formation through regulation of GATA Binding Protein 2 (GATA2) and the subsequent downregulation of TGFβ2 [141]. Moreover, it was shown recently that different pathways regulating the formation of lymphatic cord-like structures in an in vitro system can be tuned by different matrix stiffnesses together with VEGF-C concentrations [142].

Lymphatic endothelial cells express α9 and β1 integrins. More specifically, α9 integrin is necessary for ECM deposition in the valve-forming region and subsequent valve development [143]. β1 integrin is also necessary to translate the interstitial pressure to VEGF signaling in lymphatic endothelial cells to promote proliferation [144,145,146]. As described in the blood vasculature, α5β1 integrin has been shown to be involved in mechano-transduction and interacts with ALK1 and the BMP co-receptor endoglin inducing angiogenesis, suggesting a still unexplored link between α5β1 integrin and BMP signaling in lymphatic endothelial cells [87].

Several publications reported that connexin37 (encoded by *GJA4*), connexin43 (*GJA1*) and connexin 47 (*GJC2*) are produced by lymphatic endothelial cells, with major functions for connexin37 and connexin43 in lymphatic vessel development [147,148,149,150,151,152,153]. *GJA4* is induced by PROX1, FOXC2 and flow and induces, in turn, the nuclear factor of activated T cells (NFATC)/calcineurin pathway to promote valve formation in the lymphatic vessel [139,149,154]. Importantly, these genes have been demonstrated to be co-regulated by BMP9/ALK1-mediated signaling in the lymphatic endothelium, as discussed in Section 3.1, and mice deficient in BMP9 have impaired lymphatic valve development.

The adherence junction molecule VE-cadherin is particularly enriched at button-like junctions of lymphatic capillaries [155,156,157]. Just like in the vascular endothelium, VE-cadherin regulates in lymphatic endothelial cells the VEGF pathway. Moreover, VE-cadherin regulates PROX1 and FOXC2, all crucial for junction maturation, proliferation and mechano-transduction [139,158,159]. However, whether a cross-talk between BMP signaling and VE-cadherin is present in lymphatic vessels with the same functionality in permeability and angiogenesis, like in blood vessels, remains to be investigated.

### 4.5. Inhibitory SMADs (I-SMADs)

**Blood vasculature**—In addition to the R-SMADs, the inhibitory SMADs (I-SMADs) SMAD6 and SMAD7 provide an intracellular negative feedback mechanism on BMP signaling [160,161,162,163] (Figure 2; number 5). I-SMADs function as a competitive inhibitor by binding to the type I receptor, thereby preventing R-SMAD binding and activation. I-SMADs can also compete for SMAD4 binding. Moreover, an indirect mechanism of inhibition relies on the interaction of SMAD7 with Smurf ubiquitin ligases. This Smurf–SMAD7 complex degrades the R-SMADs and the type I receptors [164,165]. Several other SMAD7 mechanisms have been reported and have been extensively reviewed by de Ceuninck van Capelle et al. [166]. SMAD6 has been shown in vitro and in vivo in zebrafish to be anti-angiogenic [167]. Moreover, in mouse studies, SMAD6 protects against vessel permeability associated with changes in endothelial cell junctions and has a role in sprouting angiogenesis [163]. Recently, an additional role of SMAD6 in endothelial cell homeostasis was reported using different human endothelial cell cultures. This work showed that SMAD6 is required when vessels transition from an angiocrine state to a homeostatic maintenance state as a transducer of endothelial cell flow-mediated responses—downstream of the mechanosensory Notch1 [124]. These different effects of SMAD6 might contribute to the distinct context-dependent functions of BMP signaling in angiogenesis.

**Lymphatic vasculature**—Little information is available on the functions of I-SMADs in the lymphatic vasculature, yet SMAD7, SMAD4 and VEGF-D have been correlated with lymphangiogenesis and lymph node metastasis in patients with colon cancer [168]. High levels of either VEGF-D or SMAD7 can serve as predictors of poor prognosis and chemotherapeutic outcome in colon cancer.

### 4.6. SMAD Interacting Proteins (SIPs)

**Blood vasculature**—SMADs do not only form complexes amongst themselves but also engage in physical interactions with a large number of non-SMAD proteins [24,169] (Figure 2; number 6). SIPs include the transcriptional CoFs that confer specificity and diversity to target gene regulation by activated R-SMADs, but SIPs can also be proteins that interact with non-active monomeric SMADs. Several SIPs are also expressed in vascular endothelial cells, sometimes in a vascular bed-specific fashion. Zinc-Finger E-Box-homeobox-binding (Zeb)2 (also known as Sip1) was, for instance, recently shown to be highly expressed in liver endothelial cells, while its expression in the brain and the heart endothelium is very low [170,171]. Endothelial Zeb2 has a role in maintenance of the liver vasculature through modulation of intussusceptive angiogenesis and has a protective role against liver fibrosis upon pathological challenge [171]. The molecular mechanisms underlying its actions in the liver endothelium and whether or not these are related to BMP signaling fine-tuning remain to be determined.

**Lymphatic vasculature**—A clear link between functions of SIPs in lymphatic vessels and fine-tuning of BMP signaling herein is not yet available.

### 4.7. Interplay between BMP and TGFβ Signaling

**Blood vasculature—**The BMP and TGFβ signaling pathways have both overlapping and opposite effects because they regulate different target genes. For example, both BMP signaling and TGFβ signaling upregulate the expression of *Smad6* and *Smad7* (Figure 2, number 7). Moreover, SMAD7 subsequently inhibits both BMP and TGFβ signaling cascades, whereas SMAD6 functions only in a negative feedback loop of BMP signaling [24,162]. The opposing or balancing effects of BMP and TGFβ signaling pathways are visible in, for instance, their different regulation of the proliferation of vascular endothelial cells. Where BMP9 and BMP10 stimulate a SMAD1/5-dependent proliferation of vascular endothelial cells, TGFβ inhibits proliferation of vascular endothelial cells through pSMAD2/3 [13]. The overlapping, opposing and even antagonizing functions of BMP and especially TGFβ signaling can make investigating these pathways often challenging, and also because loss of function of one type of pathway often results in gain of function of the other type of pathway [172,173].

**Lymphatic vasculature—**Lymphatic endothelium-specific KO mice of TβRI (ALK5) or TβRII show that TGFβ signaling has a dual role: it is necessary for proper lymphatic sprouting and network formation in the dermis while restricting lymphatic endothelial cell proliferation. Moreover, TGFβ2 is the critical ligand in these processes in mice and regulates the expression of *VEGFR3* and *NRP2* in human lymphatic endothelial cell cultures [174]. Given that loss of function of TGFβ signaling often results in gain of BMP functions, it would be interesting to evaluate the BMP signaling output in these conditional TGFβ receptor KOs. Interestingly, TGFβ1 inhibits in lymphatic endothelial cells differentiated from embryonic stem cells and in HDLECs lymphangiogenesis by repressing *NR2F2* and *SOX18* encoding COUP-II and SRY (sex determining region Y)-box 18 transcription factors, and by repressing *PROX1* and *LYVE1* expression, respectively [175,176]. These findings still need to be confirmed using in vivo models.

### 4.8. Interplay between BMP and VEGF Signaling

**Blood vasculature**—The VEGF pathway is an extensively studied pathway with major functions throughout the cardiovascular and lymphatic systems. This pathway regulates processes such as endothelial cell migration, survival, proliferation, permeability, tube formation and metabolism [177]. In mammals, five secreted ligands, VEGFA, -B, -C, -D and placental growth factor, bind with different affinities to three receptors (VEGFR1, VEGFR2 and -3), with involvement of several co-receptors [177]. VEGF-A predominantly signals via VEGFR2 which has a high tyrosine kinase activity. VEGFR1 has a very low tyrosine kinase activity and acts, amongst other functions, as a decoy receptor limiting the VEGF-A-VEGFR2-mediated signaling [178,179].

In the circulatory system, numerous cooperation between VEGF signaling and BMP signaling have been reported (Figure 2; number 7). For instance, *BMP2/6/9*, but also *Bmper* and *Endoglin*, are all induced by VEGF in the endothelium, in vitro and in vivo [180]. Conversely, the development of the outflow tract in the mouse heart is controlled by BMP4/7-SMAD-mediated direct and indirect repression of *Vegf-a* [181]. Moreover, BMP9 also represses the expression of *Vegf-a* in aortic endothelial cells [41,182], and *VEGFR2* in HUVECs [47]. SMAD1/5-mediated BMP signaling is important for stalk cell identity. Moreover, SMAD1/5 represses *VEGFR2* and promotes *VEGFR1* expression [47]. BMP signaling can also transactivate VEGFR2. Indeed, the pro-angiogenic activity of BMP4 in HUVECs is mediated by a BMPRII-mediated intracellular transactivation of VEGFR2 via c-Src [183]. Recently, Pulkkinen et al. showed through in vitro and in vivo studies that BMP2, but also BMP6, signaling causes VEGFR2-mediated vessel sprouting [180].

Vascular permeability is another process that is highly dependent on VEGF and BMP pathways. VEGF-A triggers VE-cadherin internalization controlling endothelial cell permeability in a reversible manner [184]. Additionally, the VEGFR2 co-receptor NRP1 regulates endothelial barrier dysfunction, promoting permeability in a dependent or independent manner with VEGFR2 [185,186]. The BMP ligands BMP2/4/6 all increase, in a time- and dose-dependent manner, the permeability of retinal endothelial cells and HUVECs in a similar way to VEGF-A, namely, by the internalization of VE-cadherin [98]. In contrast to the permeability-inducing modalities of BMP2/4/6 and their cooperation with VE-cadherin, the BMP9/ALK1 signaling axis prevents permeability of endothelial cells. In other words, BMP9/ALK1 strengthen the vascular wall, inhibit VE-cadherin phosphorylation by VEGF signaling and stimulate the expression of occludins [187]. Overall, this illustrates that the regulation of the co-receptor stability/availability by one pathway can also indirectly regulate the other pathway. Hence, BMP–VEGF signaling interplay is dynamic in various endothelial cell types.

**Lymphatic vasculature**—The development of the lymphatic vasculature critically depends on the ligands VEGF-C and -D and their receptor VEGFR3 [188,189]. The lymphangiogenic effect of VEGF-C has been demonstrated in different mouse, zebrafish and cell culture models, all providing evidence that VEGF-C stimulates lymphatic endothelial cell proliferation, survival and migration [190,191]. Moreover, *VEGF-C* mutations have been linked to the development of lymphedema in humans [192]. Similarly, deficiency of VEGFR3 leads to lymphedema in mice and humans [189,193]. The VEGF co-receptors NRPs are also important in lymphatic development. While in the blood vasculature, NRP1 can modulate sprouting angiogenesis through VEGF-A, NRP2 enhances mostly VEGF-C binding to its receptor, VEGFR3, promoting lymphatic sprouting [185,193,194].

Despite many interactions between VEGF and BMP signaling in the vascular endothelium, little direct cross-talk in the lymphatic vasculature has been demonstrated to date. However, the earlier mentioned BMP2 and VEGF-C co-stimulation of a mouse embryoid body lymphatic differentiation model shows that BMP2-SMAD1/5/8 signaling inhibits the normally VEGF-C-mediated lymphatic endothelial cell induction [32]. Moreover, it is interesting that a combined treatment with neutralizing antibodies against ALK1 and VEGFR3 resulted in an almost complete loss of the lymphatic vasculature and an increased number of apoptotic lymphatic endothelial cells [36]. This more severe defect than when just neutralizing a single pathway suggests that ALK1 and VEGFR3 regulate different aspects of lymphatic development. It was hypothesized that inhibition of ALK1 could make the lymphatic endothelial cells more susceptible to VEGFR3 depletion and explain the more effective inhibition of lymphangiogenesis. This may open new potential therapeutic strategies in tumor metastasis where reduced lymphangiogenesis could be induced by co-inhibition of ALK1 and VEGFR3 [36].

### 4.9. Interplay between BMP and Notch Signaling

**Blood vasculature**—Notch-mediated signaling elicits—in contrast to BMP signaling—a binary type of responses, with the binding of membrane-embedded Delta-like (DLL) and Jagged (JAG) ligands and Notch receptors resulting in proteolytic processing of the Notch receptor and release of the Notch intracellular domain (NICD). The NICD effector translocates into the nucleus where it complexes with Mastermind and Recombination signal binding protein for immunoglobulin kappa J (RBP-J) to activate expression of Notch target genes such as those encoding the basic-helix-loop-helix (bHLH) proteins hairy and enhancer of split-1 (HES1), Hairy/enhancer-of-split related with YRPW motif protein (HEY) 1 and HEY2 [195].

BMP9 signaling and BMP6 signaling directly regulate the expression of *HEY2* and *JAG1* in endothelial cells through binding of pSMAD1/5 to GC-SMAD binding elements (SBE) in their promoter [196]. Moreover, pSMAD1/5/8 and SMAD4 can form a complex with NICD, resulting in BMP enhanced recruitment of the complex to the RBP-J binding site to transactivate target genes of Notch signaling such as *Hey1/2* [197]. Interestingly, several studies also indicate that pSMAD1/5/8 can activate Notch target genes such as *Hey-1/2* and *Efnb2* encoding Ephrin B2 in a Notch-independent manner [198,199,200,201] (Figure 1; number 7).

The BMP and Notch cascades interact extensively in vascular endothelial cells, amongst which there is early arterial/venous specification in the early mouse embryo, balancing the tip–stalk cell ratio in angiogenic sprouting and heart valve development [202,203]. For example, in the developing mouse heart, Notch signaling and TGFβ/BMP signaling each reciprocally trigger EndMT in the endocardium during cardiac valve formation, with *Jag1* being induced in the endocardium by BMP signaling. JAG1-Notch1 signaling regulates, in turn, *Bmp2* expression in the myocardium [93,202,203]. Additionally, loss of function of Notch and/or Alk1 signaling in zebrafish shows that both exhibit context-specific and target-specific interactions in controlling Notch target gene expression in vivo, e.g., in the dorsal aorta [198].

The BMP-pSMAD1/5/8 pathway cooperates also dynamically with Notch signaling in the establishment of a robust stalk cell phenotype during angiogenic sprouting [47,204]. In mice with an endothelium-specific deficiency in SMAD1 and -5, aberrant Dll4/Notch signaling is observed, and increased numbers of tip cell-like cells are formed at the expense of stalk cells [47]. This study revealed an intriguing synergy and antagonism of BMP and Notch signaling in the same cell type. This synergy turning into antagonism relies on a special interaction between the target genes of both pathways. HEY and HES1 are bHLH transcriptional repressors, while the ID proteins are HLH factors that can dimerize with bHLH proteins. The ID–HES1 interaction releases the negative autoregulation of HES1, which results in increased expression of *Hes1* [205]. However, upon increased production of HEY2, HEY2 can compete with HES1 for ID binding, and HEY2–ID complexes are targeted for proteasomal degradation [47,197]. Waves of relative abundance of HES1, ID and HEY components may thus pivot BMP and Notch signaling modes between synergy and antagonism [205]. Additionally, Notch also induces *Smad6* expression and thus reduces the BMP responsiveness of endothelial cells and new vessel branch formation [167].

**Lymphatic vasculature**—It is very probable that there is, just like in the vascular endothelium, a direct cross-talk between BMP and Notch signaling in the lymphatic endothelium. The Notch pathway limits the number of lymphatic endothelial progenitors in the cardinal vein [189], tempers VEGF-induced lymphatic vessel sprouting [206] and functions in lymphatic valve formation [207,208]. Interestingly, these three processes are all also dependent on intact BMP signaling, as discussed in Section 3.1 and Section 4.8. Moreover, BMP-SMAD reporter activity is observed in the vascular beds where these processes occur [62].

The molecular link between BMP and Notch functions in the lymphatic endothelium is still lacking; however, such a missing link may be TMEM100 in lymphatic endothelium specification. TMEM100 is a target gene of the BMP9/ALK1 axis in vascular endothelial cells that is also described in Section 3.1. In gain-of-function and loss-of-function mouse models of TMEM100 in the vascular endothelium, lymphatic endothelium specification is affected and Notch signaling is perturbed in both mouse models, which supports that TMEM100 controls the Notch signaling pathway [38].

It remains to be investigated whether there are functions for BMP signaling in the intestinal lacteals, the lymphatic capillaries in intestinal villi. Here, DLL4-Notch-mediated signaling promotes the regeneration of intestinal lacteals [209].

### 4.10. Interplay between BMP and WNT Signaling

**Blood vasculature**—Canonical and non-canonical WNT signaling pathways play a crucial role in cardiac development, blood vessel development and maturation [210]. The canonical WNT signaling pathway is initiated upon binding of WNT ligands to the seven-pass transmembrane Frizzled (Fzd) receptor and low-density lipoprotein receptor related protein (LRP) co-receptors [211,212]. This WNT–Fzd–LRP complex and recruited Disheveled proteins result in inactivation of the Axin destruction complex. This complex contains the scaffold protein Axin, APC, casein kinase 1 (CK1) and GSK3β. The Axin destruction complex constitutively targets the intracellular WNT effector β-catenin for proteasomal degradation in the absence of a liganded receptor. Upon WNT ligand–receptor binding, the destruction complex is inhibited, and β-catenin accumulates in the cytoplasm and translocates into the nucleus. Here, β-catenin interacts with T-cell factor (TCF)/lymphoid enhancer factor (LEF) to regulate transcription of WNT target genes [213].

WNT and BMP signaling pathways cooperate and attenuate each other in a context- and tissue-dependent manner (Figure 2; number 7). For example, in mice, BMP signaling enforces WNT signaling in the endocardium by enrichment of LEF1 through BMP-SMAD-induced production of T-Box transcription factor 20 (TBX20) [214]. In zebrafish blood vasculature, BMP signaling stimulates, via the induction of *Aggf1* encoding angiogenic factor with G-patch and FHA domains 1, β-catenin-mediated gene expression of *NR2F2* or *COUP-TFII* in the cardinal vein to promote the differentiation of venous endothelial cells. Thus, BMP signaling promotes the venous cell fate by regulating β-catenin, the main intracellular effector of the canonical WNT pathway [215].

**Lymphatic vasculature**—The canonical WNT/β-catenin signaling cascade is an exquisitely flow-sensitive pathway in the lymphatic system. WNT/β-catenin signaling is important for lymphatic and lymphovenous valve formation and contributes to patterning of lymphatic vessels in mice [216,217,218]. The activation of WNT/β-catenin signaling promotes the upregulation of WNT/β-catenin target genes, such as *Gata2*, *Foxc2*, *Connexin37*, *Integrin-a9* and *Ephrin-b2*, all genes crucial for lymphatic valve formation and lymphatic vessel morphogenesis [217,218]. These genes are also co-regulated by BMP9 signaling in lymphatic endothelial cells in cell culture [33,34]. Moreover, deficiency of β-catenin, but also of many of the previously mentioned master genes of lymphatic valve formation, results in impaired lymphatic valve development. Additionally, as described in Section 3.1, *Bmp9* KO neonates show defective valve formation. However, whether both pathways are dependent on each other in the formation of lymphatic valves has not yet been reported.

Moreover, WNT5b-dependent activation of β-catenin regulates in zebrafish embryos transcription of *Prox1a*, the major regulator of the lymphatic endothelial cell lineage. In addition, PROX1 associates with the β-catenin destruction complex and inhibits destruction of β-catenin, thereby enhancing WNT/β-catenin signaling. This supports that there is a feedback loop between PROX1 and WNT/β-catenin signaling [217,218,219]. Given that BMP9/ALK1 and BMP2 also regulate PROX1, it can be anticipated that BMP signaling may thus impact WNT signaling through PROX1.

## 5. BMP-Linked Vascular Pathologies

**Figure 3:**
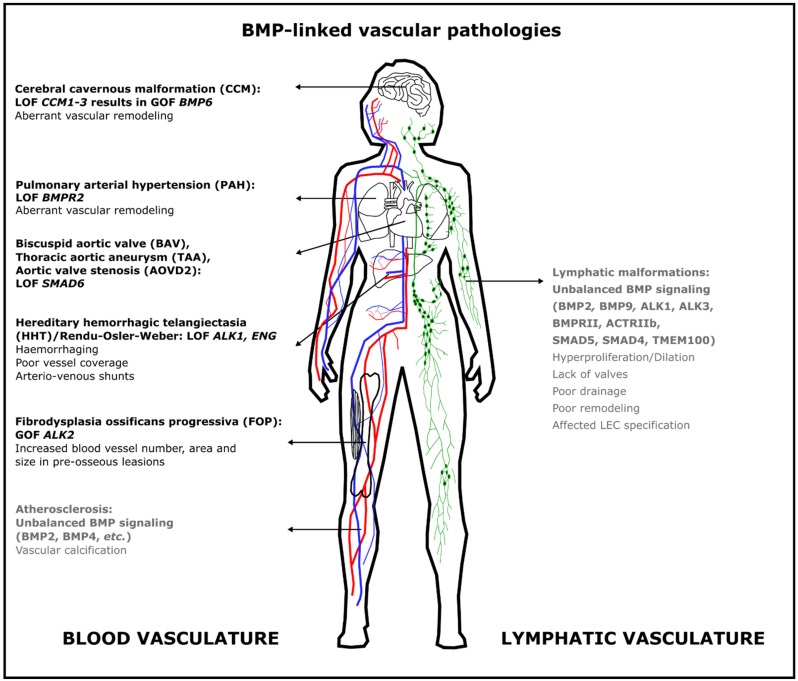
Aberrant BMP signaling in the blood vasculature causes different severe but rare diseases in humans. Some pathologies are due to loss of function of BMP signaling, whereas others result from gain of function of BMP signaling. The most frequent mutations are indicated here; additional mutations are discussed in the text. Text in gray indicates that this role for BMP signaling has only been demonstrated in animal models. Abbreviations: ACTRII: activin type II receptor; ALK: activin receptor-like kinase; AOVD2: aortic valve stenosis; BAV: bicuspid aortic valve; BMP: bone morphogenetic protein; BMPR2: BMP Type 2 receptor; CCM: cerebral cavernous malformation; ENG: endoglin; FOP: fibrodysplasia ossificans progressiva; HHT: hereditary hemorrhagic telangiectasia; GOF: gain of function; LEC: lymphatic endothelial cell; LOF: loss of function; PAH: pulmonary arterial hypertension; TAA: thoracic aortic aneurysm; TMEM100: transmembrane protein 100.

**Blood vasculature**—The germline deletion of all vascular BMP genes, except for BMP9 (*Gdf2*), and BMP receptor genes in mice causes embryonic lethality, with most prominent defects in mesoderm formation and cardiovascular development. This illustrates that this pathway exerts critical functions during embryogenesis [1,2,8]. Additionally, mutations in genes encoding BMP pathway components, ranging from ligands, type I and type II receptors, co-receptors and intracellular effectors, have been associated with cardiovascular disease [1,2,3,4] (Figure 3). Indeed, human studies have shown that impaired BMP signaling causes hereditary hemorrhagic telangiectasia (HHT), pulmonary arterial hypertension (PAH), cerebral cavernous malformation (CCM), bicuspid aortic valve with thoracic aorta aneurysm (BAC/TAA) and aortic valve stenosis (AOVD2), atherosclerosis combined with vascular calcifications and fibrodysplasia ossificans progressiva (FOP). Some of these diseases, i.e., HHT, PAH, BAC and AOVD2, result from reduced BMP signaling (loss of function), whereas others such as CCM and FOP reflect a gain of function. This illustrates, again, the critical dosage of signaling of this family of morphogens.

### 5.1. Hereditary Hemorrhagic Telangiectasia (HHT)

Patients with HHT present in specific tissues, such as the lung, brain, gastro-intestinal tract and liver, a paucity in capillaries and arteriovenous shunts/malformations (AVM). This results in a high-blood pressure blood flow from the arteries directly into the thinner-walled, less elastic veins whereby the capillary bed is bypassed. This ultimately results in enlarged blood vessels and severe hemorrhages and epistaxis. HHT is a rare disease with a frequency of about 1 in 6000 [220]. Mutations in genes encoding the co-receptor endoglin (*ENG*, MIM: 187300), the activin receptor like type 1 (*ACVRL1*, MIM: 600376) or ALK1 (*ALK1*), and occasionally also in SMAD4 (*SMAD4;* MIM: 175050), BMP9 *(GDF2;* MIM: 615506) and BMPRII (*BMPR2*; MIM: 600799), cause different subtypes of HHT [221,222,223,224,225]. In patients with HHT, the pathogenesis of AVMs involves endothelial cell proliferation, causing the primary capillary shunt to enlarge into an AVM [226,227]. Interestingly, the mutations causing HHT are all shear stress-sensitive components upstream of SMAD1/5/8 signaling and only lead to AVMs in very specific tissues. To explain the tissue specificity of AVM formation, the hypothesis of a “second hit” was proposed whereby, for example, different cooperating pathways, inflammation or mechano-sensitivity influence the site of AVM formation [228,229]. Indeed, in mice, SMAD1/5/8 signaling regulates the expression of *Gja4* (connexin37), preventing the formation of AVMs in a proliferation-independent but shear stress-regulated manner [135].

### 5.2. Pulmonary Arterial Hypertension (PAH)

Patients suffering from PAH (MIM: 178600) are characterized by an increased pressure in the pulmonary artery due to aberrant vascular remodeling of the arteries, ultimately causing heart failure [230] PAH is a rare disease with an estimated prevalence of about 15 cases per million people [231]. According to the report from the most recent World Symposium on Pulmonary Hypertension [232] germline mutations in the gene encoding BMPRII (*BMPR2*) [233,234] are detected in 70–80% of patients with a familial history of PAH, heritable PAH. An additional 10–20% of apparently idiopathic PAH cases are also caused by mutation in *BMPR2* [232].The penetrance of this severe condition is very low, with about 20% of the *BMPR2* mutation carriers further developing PAH. In a few patients, mutations have also been reported in *ACVRL1, ENG* or *SMAD9*, with *SMAD9* confusingly encoding the SMAD8 protein [53]. Overall, in all PAH conditions the balance of BMP/TGFβ signaling has been found altered, with decreased BMP signaling and increased TGFβ signaling contributing to endothelial dysfunction, vascular remodelling, inflammation and disordered angiogenesis [235,236,237,238].

### 5.3. Cerebral Cavernous Malformation (CCM)

Patients with CCM are characterized by cerebral cavernous angiomas. These are rare vascular malformations that may involve any part of the central nervous system. The prevalence of this rare disease is about 0.1% to 0.8% of the population [239]. Loss of function of any of the three *CCM* genes has been shown to induce EndMT via an exacerbation of BMP6 signaling (MIM: 116860). This gain of function of BMP signaling is a crucial event in the onset and progression of brain cavernomas, with locally increased permeability and hemorrhaging in the central nervous system [4,240].

### 5.4. Bicuspid Aortic Valve (BAV) with Thoracic Aortic Aneurysm (TAA) and Aortic Valve Stenosis (AOVD2)

Patients with BAV are characterized by an aortic valve with two rather than three leaflets. BAV is the most common congenital heart defect, with an estimated prevalence of 0.5 to 2% of the population [241], and is associated with thoracic aortic aneurysms (TAAs). BAV (MIM: 109730), also named AOVD1, is mostly caused by mutations in the gene encoding for NOTCH1, which causes a spectrum of developmental aortic valve anomalies and valve calcification diseases [242]. Frequently, BAV is an antecedent to aortic valve stenosis or insufficiency. On the other hand, AOVD2 (MIM: 614823) is mostly caused by mutations in the *SMAD6* gene. Here, in addition to the occurrence of a bicuspid aortic valve, a dilation of the ascending aorta is present [243].

### 5.5. Atherosclerosis and Peripheral Arterial Disease

Aberrant BMP signaling has also been implicated in the disease progression of atherosclerosis in vitro and in vivo. Using several pharmacological inhibitors of BMPs in in vitro and mouse studies, it was shown that BMP signaling regulates endothelial cell activation and cell differentiation in and around the atherosclerotic plaque [244,245,246]. The vascular BMP ligands BMP2 and BMP4 induce the pro-inflammatory effects in endothelial cells, leading to atherosclerotic calcification [247,248]. Moreover, an increased expression of different BMP ligands in arteries of patients with atherosclerosis associated with vascular calcifications has been observed [3,249]. Interestingly, aberrant VEGF signaling is also linked with atherosclerosis and associated arterial vascular calcifications. Whether an impaired cross-talk between BMP and VEGF pathways (Section 4.8) underlies or exacerbates this type of pathology remains to be elucidated [250,251].

While atherosclerotic obstruction is the most frequent culprit for peripheral arterial disease, the latter also triggers a rescue response whereby blood flow is redirected through remodeled collateral arteries. BMP-SMAD signaling has been shown to be involved in this shear stress-dependent remodeling response through its downstream mediator muscle segment homeobox 1 (Msx1) [246].

### 5.6. Fibrodysplasia Ossificans Progressiva (FOP)

The ultrarare autosomal dominant disease FOP (MIM: 135100, prevalence of approximately 1 in 2 million worldwide without a geographic, ethnic or gender preference) is caused by heterozygous gain-of-function mutations in the *ACVR1* gene encoding the BMP type I receptor ALK2 [252]. FOP mutations result in hyperactive SMAD1/5 signaling in response to Activin A. Patients with this severe disease develop intermittently progressive heterotopic ossifications within soft tissues, also in response to tissue trauma and surgery. Although blood vessels undergo rapid and dynamic changes in pre-osseous lesions in FOP patients, with an increased vessel number, area and size, it remains elusive to what extent the diseased vasculature contributes to the aberrant tissue repair processes [253].

**Lymphatic vasculature**—Interestingly, prominent lymphatic anomalies have thus far not been reported in the rare vascular diseases described in Section 5.1, Section 5.2, Section 5.3, Section 5.4, Section 5.5 and Section 5.6, with the exception of atherosclerosis (Section 5.6) [254].Despite the causative genetic mutation being present in all cells of the body and vasculature, all these diseases except for FOP present with an incomplete penetrance, and associated defects occur only in very specific vascular beds. It is therefore thought that a local second hit such as inflammation or altered flow patterns are required to progress to a diseased blood or lymphatic vascular bed.

Although several genetic predisposition factors for lymphedema are also target genes of BMP signaling (e.g., *KDR*, *FLT4*, *NRP2*, *GATA2*) [255,256,257], and unlike the many cardiovascular diseases that result from unbalanced BMP signaling (Figure 3), there is no direct evidence yet for a BMP involvement in lymphatic diseases in humans. However, transcriptome analysis of whole blood cells of patients with lymphatic malformations has shown that BMP signaling pathways are off balance [256]. This study does not, however, show a causative relationship between the aberrant BMP signaling levels and the disease. Nonetheless, the authors discuss that this correlative lymphatic malformation gene signature suggests, similar to the case of, for instance, CCM, a therapeutic potential of pharmacological BMP modulators in patients suffering from lymphatic malformations. Indeed, given that several genetic predisposition factors for lymphedema are also target genes of BMP signaling [33,34], and several BMP signaling modulators with different specificities are already being used in clinical studies for vascular diseases [3], this may open additional targets and strategies for BMP-based therapies for lymphatic malformations.

## 6. Conclusions

The growing wealth of single-cell transcriptome data and protein–protein interaction databases, also in lymphatic vascular cells from different niches, will progressively reveal putative additional functions of BMPs in lymphatic endothelium specification and functions. Ligand–receptor pairing between lymphatic vascular cells and cell types in their environment and analysis of target genes of BMPs will facilitate inferring the intercellular communication and BMP-sensitive niche-specific specialization of the lymphatic endothelium. In any case, it will be important to consider the context-dependent regulation of this pathway, which contributes to the subtle variations in functions of the BMP signaling components in different lymphatic endothelial cell types.

The first data on human lymphatic malformations and BMPs, together with the reported studies in zebrafish and mice, suggest a potential role for BMP signaling in human lymphatic vessel development and/or maturation. The many examples that are provided in this review on very specific BMP functions and tuning of this pathway in the vascular endothelium and the emerging picture of a cross-talk between BMP signaling and mechanobiology and VEGF, Notch and WNT signaling in the (lymphatic) vasculature can inspire the lymphatic vessel field. Moreover, the recent finding that BMP6 regulates TAZ-Hippo signaling and neo-vessel formation in the vasculature [180], as well as the growing link between BMP and vascular inflammation [115,228,229,247,248], and BMP signaling and hypoxia [53,258,259], is promising and may also plug into lymphatic vessel studies. The striking set of severe rare vascular diseases upon alterations of the BMP pathway provided in this review is likely to fuel the future exploration of this important pathway in lymphatic vessel development, physiology and pathology.

## Figures and Tables

**Table 1:** Overview of BMP signaling components that function in lymphatic vessel (LV) biology in mouse (M) and zebrafish (ZF) models or lymphatic endothelial cell (LEC) culture experiments (C).

	BMP Signaling Component	Animal Model	Function in LV	References
BMP ligands	BMP2	ZF, C, M	Anti-lymphangiogenic: restricts LEC specification	[32]
	BMP9	M, C	Anti-lymphangiogenic: Promotes LV maturation and valve maturation. Restricts LEC proliferation. Pro-lymph-vasculogenic: promotes LEC specification	[33,34,35]
BMP type I receptors	ALK1	M, C	Anti-lymphangiogenic: promotes LV maturation and remodeling	[33,34,36]
	ALK3	ZF	Pro-lymphangiogenic: promotes LEC numbers	[37]
BMP type II Receptors	BMPRII	ZF M, C	Pro-lymphangiogenic: promotes LEC numbers Anti-lymphangiogenic: promotes LV maturation and remodeling	[37] [36]
	ACTRIIB	M, C	Anti-lymphangiogenic: promotes LV maturation and remodeling	[36]
Intracellular effectors	SMAD5	ZF	Pro-lymphangiogenic: promotes LEC numbers	[37]
	SMAD4	C	Anti-lymphangiogenic: restricts LEC specification	[32]
	TMEM100	M	Anti-lymphangiogenic: restricts LEC specification	[38]

## Data Availability

Not applicable.
